# The impact of oral health disparities on smoking prevalence

**DOI:** 10.3389/fpubh.2025.1667261

**Published:** 2025-09-23

**Authors:** Pedro Henrique Carneiro, Alice Zhang, William Zhao, David M. Ojcius, Ladan Daly, Cassio Luiz Coutinho Almeida-da-Silva

**Affiliations:** ^1^Department of Biomedical Sciences, University of the Pacific, Arthur A. Dugoni, School of Dentistry, San Francisco, CA, United States; ^2^Dental Surgery Program, University of the Pacific, Arthur A. Dugoni, School of Dentistry, San Francisco, CA, United States; ^3^Department of Clinical Oral Health Care, University of the Pacific, Arthur A. Dugoni, School of Dentistry, San Francisco, CA, United States

**Keywords:** oral health, smoking, cigarettes, e-cigarettes, tobacco, inequities

## Abstract

Smoking increases the risk of heart disease, cancer, lung infections, and diabetes, and causes oral health problems. The prevalence of smoking is significantly influenced by intersecting factors such as socioeconomic status, racial and ethnic marginalization, and differing cultural practices. Importantly, minority groups experience earlier tobacco exposure and reduced access to education and cessation programs. A comprehensive analysis of individual characteristics is essential for predicting and effectively addressing tobacco-related oral and systemic health disparities. Education and prevention initiatives are key to decreasing the prevalence of smoking in populations affected by structural inequities.

## 1 Introduction

Smoking is a leading behavioral risk factor for various health issues, causing over 175 million deaths and 4–30 billion years of life lost globally from 1990 to 2021 (1). This addictive habit involves inhaling tobacco smoke with over 7,000 chemicals, including about 70 known carcinogens (2, 3). Around 40 million US adults smoke cigarettes, and nearly 4.7 million adolescents use tobacco products, mainly e-cigarettes (2, 3). Despite public health efforts, alternatives to traditional smoking, like e-cigarettes, have emerged (4, 5).

Tobacco use increases the risk of oral diseases and systemic conditions like cardiovascular disease and cancer (2, 3, 6, 7). It harms overall health, causes inflammation, impairs immune function, and affects embryonic development (2). Secondhand smoke is linked to cancer and respiratory and cardiovascular diseases (2). Although e-cigarettes are often marketed as safer than combustible tobacco, evidence indicates that they still pose measurable risks to oral health (8).

This review advances the central argument that oral health disparities are not only consequences of tobacco use but also function as mediators and amplifiers of smoking-related health outcomes. While the biological toxicity of tobacco products has been well described, less attention has been given to how these effects intersect with social determinants of health. Discrimination, inequality, and limited access to care disproportionately affect minority and underserved groups, shaping both tobacco exposure and oral health vulnerability (9). By integrating biological mechanisms with structural and cultural factors, this review highlights oral health as a critical but underexplored pathway linking tobacco use to broader health inequities.

**Figure 1:**
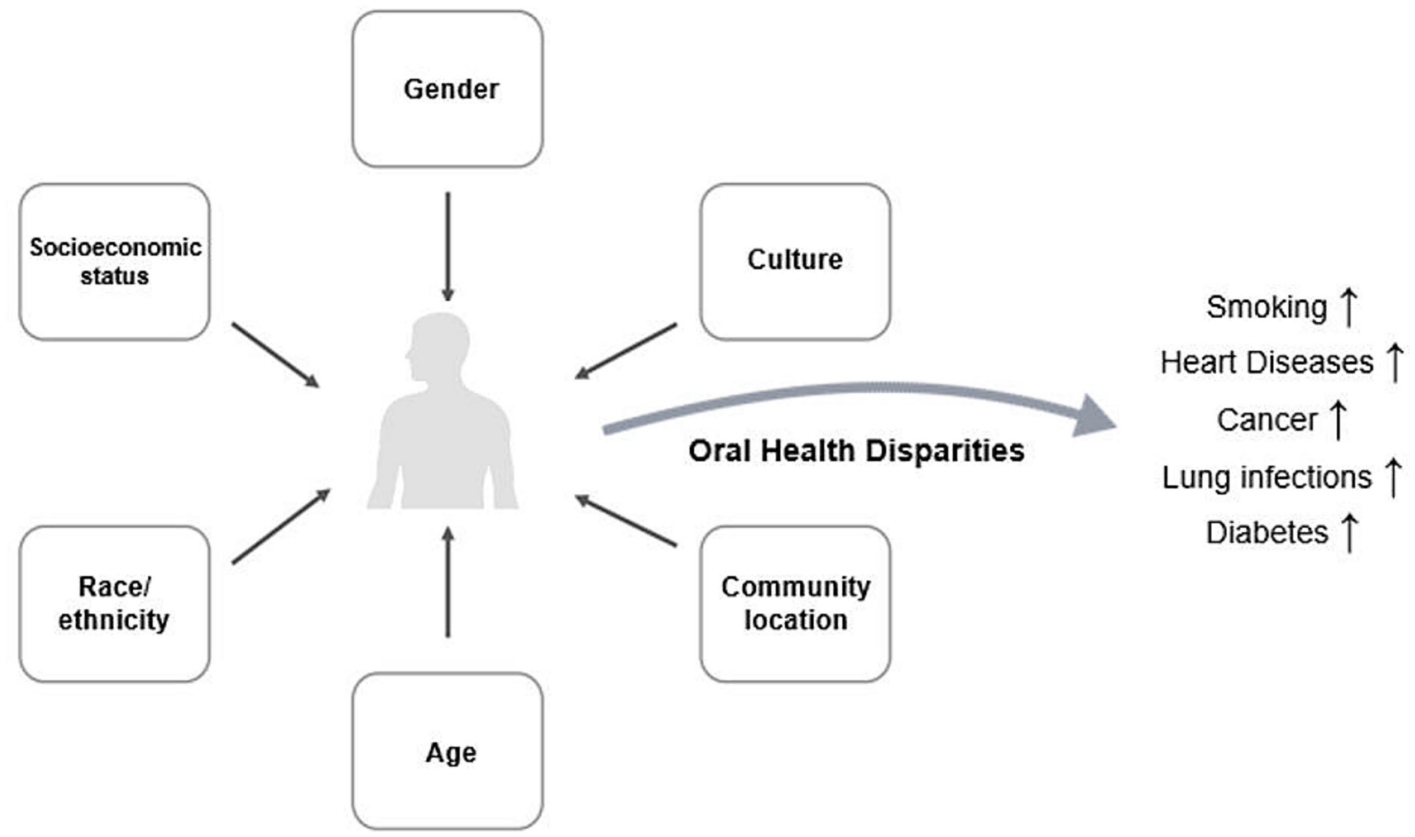
Conceptual model of tobacco-related oral health disparities. Social determinants of health shape disparities in oral health, which in turn influence tobacco behaviors such as smoking, e-cigarette use, and cessation challenges. These behaviors contribute to both oral and systemic health consequences, including increased morbidity and mortality.

Health disparities, rooted in discrimination and inequality, disproportionately affect minority groups and contribute to significant differences in oral health (9), as represented in Figure 1. These inequities magnify the harms of tobacco use, compounding risks at both biological and social levels. Prevention and education remain essential, as smoking damages nearly every organ system (2, 10). Importantly, tobacco-related risks also extend to non-smokers through secondhand exposure, adding billions of dollars in healthcare costs and lost productivity each year (11, 12).

Understanding smoking through the lens of oral health disparities reframes tobacco’s impact as both a biological insult and a social amplifier of disease. Factors such as socio-economic status, race, gender, culture, and geographic residency influence smoking patterns and related outcomes (13). Ethnic minorities and rural populations often face higher stress and fewer resources, compounding risks (14), while cultural norms may reinforce tobacco use as a social activity or coping strategy (15). Recognizing these overlapping vulnerabilities underscores the urgency of prevention and education efforts, not only to reduce smoking prevalence but also to mitigate the disproportionate burden carried by at-risk populations. In this way, this review positions oral health disparities at the center of tobacco-related disease research and public health policy.

To identify relevant studies for this review, a systematic literature search was conducted using keywords including combinations of terms related to “tobacco,” “cigarette,” “e-cigarette,” “vaping,” “oral health,” “periodontitis,” “health disparities,” “social determinants,” and “smoking cessation.” Inclusion criteria were: original research, systematic reviews, or meta-analyses published in English from 2000 onward, focusing on tobacco exposure and oral or systemic health outcomes, with attention to social determinants or population disparities. Studies were excluded if they were case reports, or not directly addressing tobacco-related health impacts.

## 2 Factors affecting smoking rates and health

This review is guided by the social determinants of health and intersectionality frameworks. The social determinants model emphasizes how structural factors—such as socioeconomic status, education, and healthcare access—shape both tobacco use and oral health outcomes. Intersectionality highlights that these determinants rarely act in isolation; instead, overlapping identities such as race, gender, and location interact to create compounded vulnerabilities (Figure 1). By applying these frameworks, we structured our study to show not only the biological effects of tobacco but also how social and structural inequities amplify oral health disparities. This approach provides oral health disparities as central mediators of tobacco-related disease burden.

### 2.1 Socioeconomic status

Socioeconomic status (SES), typically measured by income, education, and occupation, is a key determinant of both tobacco use and oral health outcomes. Individuals with lower SES often face a disproportionate burden of risk factors, including limited access to preventive services, reduced awareness of health risks, and greater exposure to environments where tobacco use is normalized (16). These conditions contribute to persistent health disparities and exacerbate the global burden of tobacco-related diseases.

Education levels also correlate with smoking prevalence. In countries like India, Iran, South Korea, Sweden, and the Czech Republic, lower education levels are associated with higher smoking rates and lower cessation rates (17). Adults with lower educational attainment and income levels are significantly more likely to smoke, less likely to attempt cessation, and less successful when they do (17, 18). Limited access to cessation programs, pharmacological aids, and healthcare coverage further compounds these disparities (19–21). As a result, tobacco use among disadvantaged groups imposes disproportionate health and economic costs, perpetuating cycles of inequality. Studies show higher smoking prevalence in lower socioeconomic groups.

In low- and middle-income countries, the SES–tobacco relationship shows a different and more complex pattern. For example, in China, smoking prevalence is often higher among wealthier, more educated adults compared to their lower-income counterparts, which contrasts with patterns in high-income countries (17, 22). Meanwhile, in South Asia, tobacco use is strongly concentrated in poorer populations, contributing to alarmingly high levels of periodontal disease; an Indian survey reported periodontitis in over 70% of adults, particularly among low-income tobacco users (23). In Eastern Europe, dentists highlight socioeconomic barriers to adequate periodontal risk management, while in Sub-Saharan Africa, reviews emphasize that structural inequities, such as the absence of biobanks and diagnostic infrastructure, further limit progress (24, 25).

These findings underscore that SES shapes tobacco use and oral health outcomes differently across global contexts. In high-income countries, smoking is increasingly concentrated in marginalized populations, while in low- and middle-income countries the association is shaped by aggressive tobacco industry marketing and diverse cultural and economic dynamics. Addressing these disparities requires context-specific policies, including tailored cessation interventions, increased investment in oral health infrastructure, and stronger regulatory measures to counter tobacco industry targeting in vulnerable populations.

### 2.2 Age

Age is a critical determinant of smoking behavior and its oral health consequences. Tobacco initiation typically occurs in adolescence or early adulthood, while cumulative exposure increases the severity of periodontal disease and oral cancers later in life (26). Younger populations are particularly vulnerable to peer and environmental influences, while older adults often face compounded risks due to lifelong exposure and comorbidities (9, 27).

Tobacco use is disproportionately concentrated among young adults in England, with nearly 90% of smokers starting before the age of 18 (16). Recent trends show an alarming rise in e-cigarette use among adolescents, which may act as a gateway to combustible tobacco (28). Risk behaviors during adolescence, such as lack of parental supervision, exposure to smokers, low self-esteem, and depressive symptoms, increase tobacco use (26). Foster care environments, which are less structured, contribute to higher smoking rates and risky smoking practices among youth (29).

Patterns of tobacco use by age in low- and middle-income countries vary but often reflect early initiation and long-term exposure. In Southeast Asia, adolescents show some of the highest global rates of smokeless tobacco use, particularly in low-income settings (30). In Sub-Saharan Africa, tobacco use among youth is rising due to targeted industry marketing and weak regulatory enforcement. Meanwhile, in Eastern Europe, surveys reveal high lifetime prevalence of smoking, with initiation often occurring in early adolescence, contributing to elevated rates of oral cancer later in life (31).

Age influences tobacco-related oral health risks across the lifespan, with early initiation and cumulative exposure driving long-term harm. Prevention policies targeting youth in both high- and low-income contexts are essential. Strengthening school-based education, regulating e-cigarettes, and expanding surveillance systems are crucial steps to mitigate generational cycles of tobacco-related oral disease.

### 2.3 Race and ethnicity

Race and ethnicity are important social determinants of health that intersect with tobacco use and oral health disparities (29, 32). Differences in cultural norms, socioeconomic status, access to healthcare, and targeted industry marketing contribute to variations in tobacco prevalence and oral disease burden across racial and ethnic groups (29, 32).

In the US, Black individuals who smoke experience greater nicotine dependence and have less success in quitting smoking than non-Black individuals (33). Despite smoking fewer cigarettes per day on average, they face a higher risk of developing tobacco-related diseases, likely due to structural and environmental factors such as targeted marketing and limited access to cessation resources (34). Differences in biomarkers of smoke exposure and nicotine intake levels may also explain this disparity (34). The tobacco industry often uses predatory marketing techniques to target non-White ethnic groups. Black young adults are three times more likely to use flavored cigars and more likely to smoke menthol-flavored cigarettes (35, 36). The increased use of flavored tobacco products by Black individuals has been shown to impact oral health to a higher degree (37). While educational attainment generally reduces smoking prevalence, high-income Chinese-Americans continue to experience elevated smoking rates, suggesting that the protective effects of socioeconomic status may be smaller for historically marginalized groups (38, 39).

In low- and middle-income countries, ethnic and minority groups often carry a disproportionate burden of tobacco-related oral disease. In South India, populations show higher prevalence of smokeless tobacco use, with significant oral precancerous lesions (40). In Sub-Saharan Africa, ethnic minorities in rural areas report limited access to oral healthcare services, contributing to high untreated disease rates (41, 42).

Race and ethnicity shape both exposure and vulnerability to tobacco-related oral health harms. Systemic inequities and targeted marketing perpetuate disparities, while ethnic minorities face structural barriers to care. Addressing these inequities requires culturally sensitive prevention and cessation strategies, regulation of targeted tobacco marketing, and improved integration of oral health services into primary care.

### 2.4 Culture

Culture strongly influences tobacco use behaviors and oral health practices. Norms, beliefs, and traditions can either promote tobacco consumption or discourage it, shaping patterns of initiation, cessation, and long-term outcomes (43, 44). Cultural acceptance of tobacco often overlaps with socioeconomic and policy environments, reinforcing disparities.

First-generation immigrants often have lower mortality rates than US-born individuals from similar racial/ethnic backgrounds, attributed to healthy cultural practices and lower smoking prevalence (45). Immigrants are 52% less likely to smoke compared to US-born individuals (45). However, acculturation may lead to higher smoking rates among some immigrant populations in the US (46, 47). The “Healthy Immigrant Effect” describes how immigrants’ health outcomes worsen over time in the US, particularly among female immigrants and those arriving before age 13 (45). Immigrants face barriers to healthcare and smoking cessation treatment, such as language barriers and lack of insurance (47).

Cultural norms play a central role in low- and middle-income countries. In South Asia, chewing tobacco, betel quid, and areca nut remain widely accepted practices, often tied to tradition and social gatherings (48). In parts of Sub-Saharan Africa, tobacco use is integrated into rituals and community life, while in Eastern Europe, smoking has long been embedded in social culture, especially among men, reinforcing its normalization (49, 50). These cultural practices amplify the burden of oral disease by delaying recognition of risks and undermining public health messaging.

Cultural drivers of tobacco use represent both barriers and opportunities for intervention. Public health campaigns must be culturally adapted to address traditions without stigmatizing communities.

### 2.5 Urban vs. rural communities

An individual’s living location—specifically the divide between urban and rural communities—profoundly shapes tobacco use and oral health outcomes. Rural populations often experience higher tobacco prevalence, reduced access to care, and worse oral health outcomes compared to urban populations (22). These disparities are compounded by socioeconomic and infrastructural challenges.

As smoking has become less socially acceptable in most of the US, rural communities, especially those economically reliant on tobacco, may still view it positively (51, 52). While US smoking rates have declined, rural populations show slower or no reduction compared to urban areas (51, 53–55). Rural residents are less likely to participate in or succeed in smoking cessation treatments, which occur three times less often in rural primary care clinics (51, 53, 54, 56). Barriers include fewer resources, healthcare providers, and clinics, as well as lower health insurance prevalence (51, 53, 54).

Rural–urban disparities are even more pronounced in low- and middle-income countries, where rural residents often lack access to oral health infrastructure and preventive services, contributing to high rates of untreated periodontal disease (49). In Eastern Europe, urban areas may have a lower prevalence of smoking but better access to cessation services, leaving rural residents disproportionately vulnerable (25, 50).

The urban–rural divide reflects how geography intersects with access, culture, and industry influence. Bridging these disparities requires scaling up community-based cessation programs, expanding oral health workforce distribution, and strengthening rural health infrastructure.

### 2.6 Gender

Gender dynamics significantly shape tobacco use and oral health outcomes, with differences driven by social norms, industry targeting, and biological susceptibility. Historically, due to the lack of social acceptability, women did not engage in smoking until several decades after men began smoking cigarettes (57). Generally, the prevalence of smoking differs between genders based on age group and stage in life. As adolescents, males and females tend to have similar levels of smoking prevalence, with adolescent girls having slightly higher levels because of social factors such as concerns over thinness or as a means of dieting (57). During early and middle adulthood, more psychosocial elements come into play and change the prevalence of smoking to be higher in men than women (58).

In many cultures around the world, smoking is perceived as a normal and typical aspect of masculine behavior, compared to a wayward act with negative connotations in women, which impacts prevalence. For example, in Japan, the percentage of male smokers is more than three times the percentage of female smokers (59). Conversely, in an environment where conformity to social and cultural norms holds less weight, such as the US, smoking prevalence between genders is more even, with less than a 6% difference between men and women (59).

There is a marked difference between genders when considering cessation of smoking, with women often having less long-term success initially quitting and maintaining cessation than men (60). A variety of environmental, social, and psycho-pharmacological factors contribute to this, including a difference in income and poverty rates between genders (15, 60). One notable qualifier to this trend is parenthood. Women with one child are more likely to quit smoking than childless women of a similar age, and these odds increase for each additional child up to 3 children (60). Men with children also experience greater chances of cessation, but these odds are consistent and do not differ with more children (60). Additionally, because women are more likely to expect smoking to contribute to weight control, a slower smoking cessation rate is seen in women compared to men over time (15).

Individuals who are part of a gender minority (such as nonbinary, genderqueer, intersex) were found to be more likely than cisgender individuals to use assistance in their cessation attempts. However, more research is needed to determine the smoking prevalence and cessation success for gender minority populations (61).

Gender shapes not only the prevalence of tobacco use but also the cultural and biological vulnerabilities to oral disease. Gender-sensitive policies, including targeted prevention for women and culturally adapted interventions, are crucial to addressing these emerging disparities.

## 3 Discussion

While studies have examined how specific socio-cultural groups are affected by smoking, comprehensive analyses of individual identity factors—such as gender, culture, and socioeconomic status—and their relationship to smoking behaviors remain limited. This review identifies critical gaps in understanding why certain groups have higher smoking prevalence and the resulting adverse health impacts, particularly in relation to oral health.

**Figure 2:**
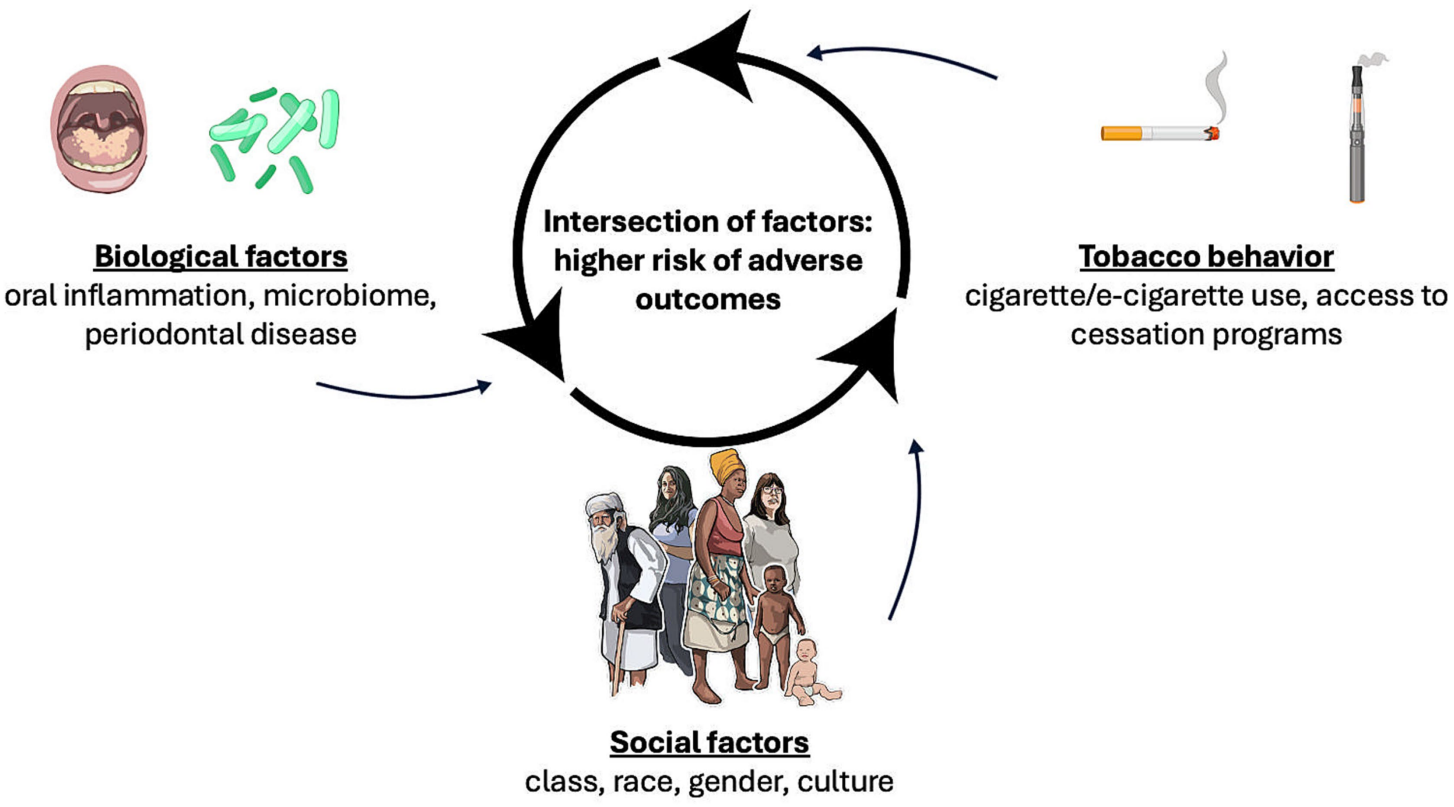
Biological and social factors interaction underlying tobacco-associated oral health disparities. A network illustrates how biological mechanisms (oral inflammation, microbiome alterations, periodontal disease), social factors (class, race, gender, cultural background), and tobacco use behaviors (smoking, e-cigarette use, cessation access) interact. Intersecting vulnerabilities magnify adverse health outcomes.

Increased smoking incidence is often linked to the failure or absence of targeted smoking cessation programs, with societal segments showing higher prevalence also experiencing lower cessation efforts. Smoking significantly affects oral health, increasing the risk of periodontal disease, altering oral microbiome diversity, and potentially amplifying systemic inflammation (Figure 2).

All health disparities are interconnected, and individuals may experience multiple oral health inequities simultaneously, highlighting the complexity of these disparities and their impact on smoking prevalence (Figure 2). For instance, an individual’s risk of developing a tobacco-related disease may be influenced by their age and culture, but not necessarily by their gender or socioeconomic status. Future research should integrate multidimensional assessments of oral health disparities, medical history, and living environment to fully capture how these factors interact with smoking behaviors and disease risk.

Socioeconomic status and racial/ethnic and cultural identities emerge as the most influential determinants of smoking prevalence. Minority individuals in these groups often face earlier tobacco exposure, limited access to education and cessation programs, and culturally distinct smoking practices, contributing to inequities in tobacco-related oral health. While age, gender, and urban versus rural residence also shape these disparities, there remains a pressing need for longitudinal studies to track smoking behaviors and oral health outcomes over time, for culturally tailored interventions targeting high-risk populations, and for research exploring the intersection of oral health disparities and tobacco use across diverse populations and settings. Particular emphasis should be placed on studies in low- and middle-income countries and among adolescents, where current data are limited.

While tobacco use is consistently associated with adverse oral health outcomes, including periodontal disease and altered microbiome diversity, the majority of evidence comes from cross-sectional and observational studies. These studies can identify correlations but cannot definitively establish causality. Therefore, it remains unclear whether tobacco use directly causes oral health deterioration or whether other confounding factors—such as socioeconomic status, cultural practices, and access to dental care—contribute to these observed outcomes. Future studies are needed to clarify the causal pathways linking smoking behavior to oral and systemic health impacts.
